# Efficacy of N-acetylcysteine in enhancing paracetamol's analgesic effect and inhibiting hepatotoxicity: a preclinical mouse model

**DOI:** 10.1097/PR9.0000000000001461

**Published:** 2026-07-16

**Authors:** Basile Chrétien, Romain Goy, Mickaël Guillaume, Guillaume Bazin, Mathilde Collet, Jean-Jacques Dutheil, Catherine Baugé, Denis Vivien, Véronique Agin, Charles Dolladille

**Affiliations:** a Pharmacology Department, Caen University Hospital, Caen, France; b Neuropresage Team, Normandie University, UNICAEN, INSERM, UMR-S U1237, Physiopathology and Imaging of Neurological Disorders (PhIND), Institute Blood and Brain @ Caen-Normandie (BB@C), GIP Cyceron, Caen, France; c Normandie University, UNICAEN, INSERM, UMR-S U1237, Physiopathology and Imaging of Neurological Disorders (PhIND), Institute Blood and Brain @ Caen-Normandie (BB@C), GIP Cyceron, Caen, France; d Experimental Stroke Research Platform, Normandie University, CURB, Caen, France; e Department of Clinical Research and Innovation, Caen University Hospital, Caen, France; f Normandie University, UNICAEN, UR7451 Bioconnect, Caen, France; g Normandie Univ, UNICAEN, INSERM, ANTICIPE, U1086, Caen, France

**Keywords:** Analgesia, Hepatoprotection, N-acetylcysteine, Pain, Paracetamol

## Abstract

**Introduction:**

Paracetamol (PCM) is extensively administered in clinical settings to alleviate mild to moderate pain and/or decrease fever. However, noncompliance with prescribed dosages can lead to severe and irreversible liver damage from PCM overdose. Although N-acetylcysteine (NAC) serves as the standard antidote for PCM toxicity, no commercially available combination of PCM and NAC has been marketed to mitigate its toxic effects. **Objectives: **This study aims to evaluate both the antinociceptive and hepatoprotective effects of the PCM and NAC combination in a murine model.

**Methods:**

Male and female C57Bl6/J mice were assigned to 4 groups. Group A received oral PCM exclusively, administered every 6 hours (4 doses within 24 hours), whereas groups B-D were administered PCM alone, a combination of PCM/L-NAC (N-acetyl-l-cysteine), or PCM/D-NAC (N-acetyl-d-cysteine) every 4 hours (6 doses within 24 hours).

**Results:**

Both PCM/L-NAC and PCM/D-NAC combinations yielded superior analgesia compared with the standard regimen. Furthermore, L-NAC administration mitigated the increase of glutamate pyruvate transaminase, a key hepatic marker associated with PCM hepatotoxicity. In addition, a protective effect from D-NAC in conjunction with PCM on glutathione levels was suggested. Our findings suggest that NAC enhances the analgesic properties of PCM while concurrently reducing its hepatotoxic potential in mice. Moreover, data indicate that L-NAC may represent a more effective candidate than D-NAC for this purpose, particularly regarding hepatoprotection.

**Conclusion:**

This study presents novel insights into the synergistic effects of the PCM/NAC combination on pain relief and hepatic safeguarding, warranting consideration for the development of novel therapeutic strategies targeting pain and fever management in patients.

## Abstract 2

Supplemental Digital Content is Available in the Text.

Synergistic coadministration of paracetamol and N-acetylcysteine in mice enables safer high-frequency dosing, yielding superior analgesia and hepatoprotection compared with standard regimens.

## 1. Introduction

Paracetamol (PCM), or acetaminophen, is a prevalent medication used for the management of fever and pain across diverse age groups and clinical contexts.^45^ The recommended maximum dosage for adults varies by country but typically does not exceed 4 grams per day, administered in doses of up to 1 gram.^38^ However, this upper limit is underscored by the risk of toxicity, predominantly affecting the liver, which can manifest at elevated dosages.^7^ Importantly, the pharmacokinetics of PCM at the 4-gram daily dosage may result in intervals of diminished efficacy,^13^ given that PCM efficacy is dose-dependent.^10^ During such periods, additional medications, including nonsteroidal anti-inflammatory drugs (NSAIDs)^43^ and opioids, are often used to manage fever or pain. Regrettably, NSAIDs exhibit a comparatively unfavorable safety profile relative to PCM^15,16^ and are contraindicated in specific countries when bacterial infections are diagnosed, because of their risk of inducing severe complications, including death.^1,46,47^

Conversely, although opioids are effective in managing intense pain, they entail substantial risks, such as addiction, respiratory depression, and the potential for overdose.^3,20,22^

Only one study has examined the safety and pharmacokinetics of increasing doses of PCM in humans.^10^ Nevertheless, the challenge of prescribing higher PCM doses safely, while mitigating associated toxicity, remains a critical clinical concern.

N-acetylcysteine (NAC) is the standard antidote for PCM overdose, primarily by replenishing glutathione reserves and neutralizing the toxic metabolite N-acetyl-para-benzoquinone-imine (NAPQI).^23^ Importantly, NAC's mechanism of action does not compromise the therapeutic efficacy of PCM. Despite this, to our knowledge, no commercially available combination of PCM and NAC has been marketed with the intent of increasing the daily PCM dosage for enhanced management of fever and pain. To date, the scientific focus on PCM/NAC combinations has predominantly been directed towards preventing hepatic toxicity in instances of PCM overdose, as opposed to investigating the potential for NAC to facilitate increased daily PCM dosages. A combination of PCM and methionine (another antidote) was previously marketed in the United Kingdom to mitigate toxicity after overdose. However, the commercial success has been severely limited because of concerns regarding cost, and the poor gastrointestinal tolerance associated with methionine.^42^

This study aims to bridge this gap by using a murine model to illustrate that the PCM and NAC combination, administered in a regimen of 6 doses per-24 hours, achieves superior analgesic efficacy over time relative to the conventional regimen of 4 doses within the same timeframe, while concurrently providing protection against hepatic toxicity. Furthermore, this investigation endeavors to compare the effects of N-acetyl-l-cysteine (L-NAC) and N-acetyl-d-cysteine (D-NAC). It is widely recognized that only L-NAC undergoes conversion to glutathione, thus exhibiting antioxidant capabilities against NAPQI.^9^ Nonetheless, both D-NAC and L-NAC possess intrinsic antioxidant properties irrespective of their conversion to glutathione.^29^ This comparative analysis is vital, as it may unveil distinct effects on hepatic protection and analgesic efficacy, informing the optimal application of NAC enantiomers in clinical practice.

## 2. Materials and methods

### 2.1. Study design

This study evaluated the efficacy of the PCM and NAC combination in alleviating pain and preventing hepatic toxicity in mice. A controlled experimental design was used, comprising 4 treatment groups and involving a total of 80 C57Bl6/J mice.

### 2.2. Animals and ethical considerations

Experiments were conducted on 10- to 12-week-old male and female mice (males: 25–30 g; females: 18–22 g) obtained from Janvier Labs (Le Genest-Saint-Isle, France). Mice were housed in standard polypropylene cages (22 × 37 × 19 cm, Charles River, L'Arbresle, France; 5 mice per cage) with access to food and water ad libitum, in a room with regulated temperature and light (temperature maintained at 23°C ± 2°C, 12-hour light/12-hour dark reverse cycle) (Supplementary Method 1, http://links.lww.com/PR9/A434).

### 2.3. Experimental groups

Mice (n = 80) were randomly distributed into 4 independent groups (20 per group; 10 males and 10 females per subgroup) as follow (Supplementary Methods 2, http://links.lww.com/PR9/A434).

#### 2.3.1. Group A

Classic PCM Scheme Group: 100 mg/kg of PCM, administered every 6 hours.

#### 2.3.2. Group B

High Dose PCM Group: 100 mg/kg of PCM, administered every 4 hours.

#### 2.3.3. Group C

PCM + L-NAC Group: 100 mg/kg of PCM +100 mg/kg of L-NAC, administered every 4 hours.

#### 2.3.4. Group D

PCM + D-NAC Group: 100 mg/kg of PCM +100 mg/kg of D-NAC, administered every 4 hours.

### 2.4. Procedure

**Figure 1.**
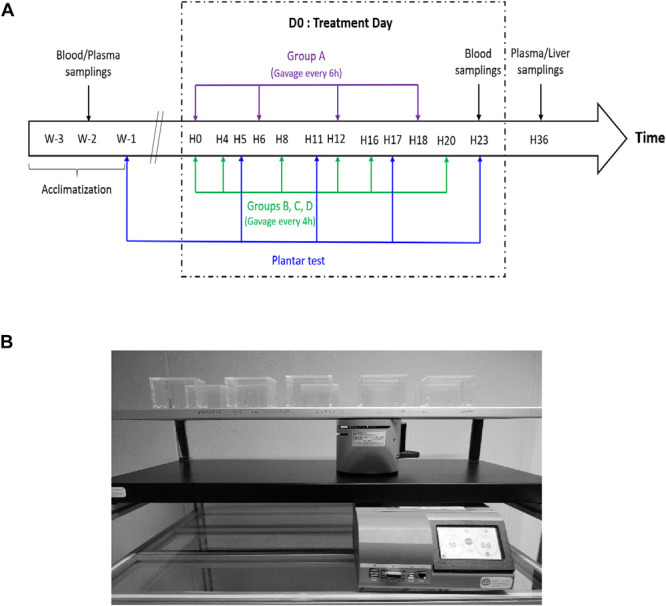
(A) Timeline of the experimental procedure. Arrows indicate oral administration of treatments (purple and green arrows) and behavioral tests (blue arrows). Group A: 100 mg/kg of paracetamol (PCM), every 6 hours; group B: 100 mg/kg of PCM, every 4 hours; group C: 100 mg/kg of PCM + 100 mg/kg of N-acetyl-l-cysteine (L-NAC), every 4 hours; group D: 100 mg/kg of PCM + 100 mg/kg of N-acetyl-d-cysteine (D-NAC), every 4 hours. (B) Image showing the experimental setup: the plantar test. D, day; H, hour; W, week.

The procedure is outlined in Figure 1.

#### 2.4.1. Assessment of pain

Before assessment, mice were acclimated to the animal care facilities and handling over a period of 3 weeks, with visits occurring every 2 days, excluding cage change days, to minimize stress. All procedures were conducted during the animals' dark cycle (between 7:00 and 19:00) by researcher who was blinded to the experimental treatments.

##### 2.4.1.1. Habituation

Mice were habituated to the testing chamber for 2 days before measurements (W-1: D-3, D-2; Fig. 1A), for 15 minutes each day.

##### 2.4.1.2. Apparatus

A plantar test (Hargreaves apparatus; Ugo Basile; Salon de Provence, France) adapted from^5,8,39,44^ was used to assess thermal sensitivity to noxious radiant heat and its evolution over time (Fig. 1B).

##### 2.4.1.3. Pain threshold measurement

After habituation, the sensitive-discriminative dimension of thermal pain was assessed using the radiant heat source (infrared light source), set at 0.5 cm below the glass plate, and calibrated to emit a consistent thermal flux at 50% of its full capacity (50%: 90 mW/cm^2^; full capacity: 180 mW/cm^2^). Thermal stimuli were applied through the glass floor to the plantar surfaces of the right and left hind paws. Paw withdrawal latencies were recorded, with a minimum cutoff of 2 seconds and a maximum of 15 seconds. Stimulation was automatically ceased after 15 seconds (cutoff time) to prevent any potential tissue damage. Each hind paw was tested 3 times with intervals of 3 to 4 minutes between trials. The final thermal withdrawal latency was derived from the mean of the 3 measurements. These measurements were conducted at D-1 (baseline) and at 6-hour intervals on the treatment day (D0: H5, H11, H17, H23; Fig. 1A).

#### 2.4.2. Drug administration

Paracetamol alone (CliniSciences, Nanterre, France), or in combination with L-NAC (CliniSciences) or D-NAC (CliniSciences), was solubilized in the water from the mice home bottles and administered orally (gavage). Group A received treatment every 6 hours (ie, 4 administrations; Fig. 1A), whereas groups B-D received treatments every 4 hours (ie, 6 administrations; Fig. 1A). Before the commencement of experiments, all animals underwent a 16-hour fasting period.

#### 2.4.3. Biochemical measurements

##### 2.4.3.1. Blood collection

Animals were subjected to deep anesthesia with 5% isoflurane, followed by maintenance with 2% isoflurane in a 70%/30% NO_2_/O_2_ mixture for a few minutes. Blood was drawn from the mandibular vein using a lancet (sodium citrate 0.129 M; at a ratio of 1:9 of the blood volume collected). The samples were subjected to centrifugation twice at 1500 g for 15 minutes at room temperature to separate the plasma and obtain platelet-poor plasma.

##### 2.4.3.2. Analyses

Samples were immediately placed into liquid nitrogen after collection and stored at −80°C until analysis. Both free (Glutathione [Reduced] [GSH]) and oxidized (Glutathione [Oxidized] [GSSG]) glutathione were monitored via liquid chromatography with fluorescent detection (Shimadzu Nexera 40 Series) following the recommendations of the Glutathione in Whole Blood Chromsystems kit (order no: 66000). Heparinised plasma aspartate aminotransferase (AST) and alanine aminotransferase (ALT) concentrations were analyzed using spectrophotometry on the Alinity C system (Abbott). Details on the analyses are available in the supplementary materials, http://links.lww.com/PR9/A434.

### 2.5. Outcome measures

#### 2.5.1. Primary outcome

Pain Assessment: The principal endpoint was the nociceptive threshold, measured via the plantar test. The latency for paw withdrawal was systematically recorded and analyzed.

#### 2.5.2. Secondary outcomes

Hepatic function was assessed through biochemical assays measuring GSH, GSSG, ALT, and AST concentrations.

### 2.6. Exclusion criteria

Mice displaying dominance conflicts among conspecifics (resulting in injuries) or errors in treatment administration were excluded from behavioral analysis (group A, n = 2; group B, n = 1; group C, n = 1; group D, n = 2). Furthermore, the following criteria were used to exclude samples from biochemical analyses: overt mechanical damage or disruption during biological sample preparation, rendering the sample unsuitable (group A, n = 6; group B, n = 5; group C, n = 4; group D, n = 3).

### 2.7. Statistical analysis

Statistical analyses were conducted using GraphPad Prism software (version 10.2.2). The distribution of samples was evaluated using Shapiro–Wilk W tests. Because of the non-normal distribution of the data, nonparametric statistical tests were applied for all analyses. Friedman tests were used for intragroup multiple comparisons. In instances of significance, differences from baseline were computed using Wilcoxon signed-rank tests for matched samples. Intergroup multiple comparisons were performed using Kruskal–Wallis tests. When significant, Dunn tests were executed as post hoc analyses. The statistical significance of differences between the 2 PCM-groups was examined using Mann–Whitney U tests. An alpha level of *P* < 0.05 was established for determining significance in all statistical tests; all tests were two-tailed. Male and female data were pooled, as no significant sex differences were observed in analgesic or hepatoprotective effects.

## 3. Results

**Figure 2.**
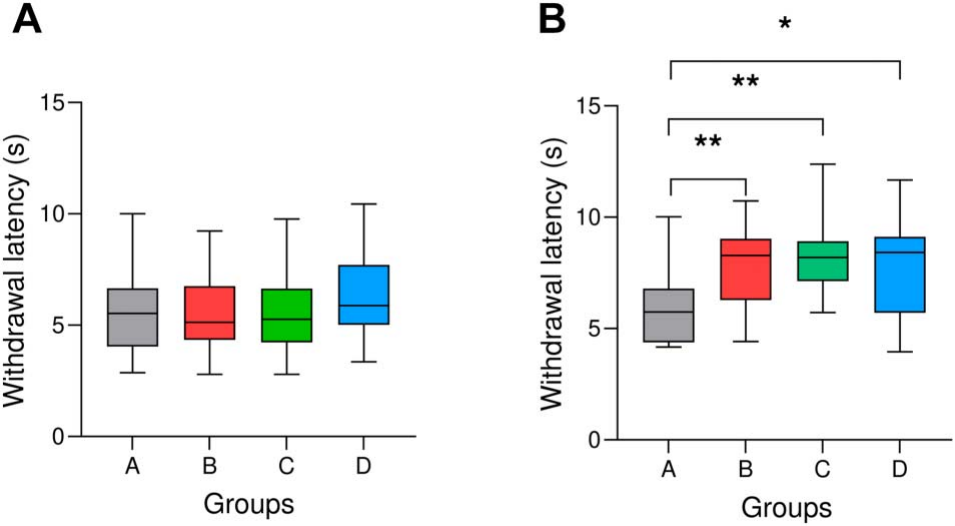
Paw withdrawal latencies in the plantar test. (A) Similar paw withdrawal latencies before treatments (baseline; Kruskal–Wallis test: *P* > 0.05). (B) Antinociceptive effect of PCM alone and in combination with N-acetyl-l-cysteine (L-NAC) or N-acetyl-d-cysteine (D-NAC) at 17 hours post-treatment. Group A (n = 18): 100 mg/kg of PCM, every 6 hours; group B (n = 19): 100 mg/kg of PCM, every 4 hours; group C (n = 19): 100 mg/kg of PCM + 100 mg/kg of L-NAC, every 4 hours; group D (n = 18): 100 mg/kg of PCM + 100 mg/kg of D-NAC, every 4 hours. Kruskal–Wallis test: *P* < 0.01. Dunn tests: *P < 0.05; **P < 0.01. Boxplots show distributions with black horizontal lines indicating the median, box margins denoting the lower and upper quartiles, and the whiskers extending from the box out to the most extreme observation within 1.5 times the interquartile range from the box. PCM, paracetamol.

**Figure 3.**
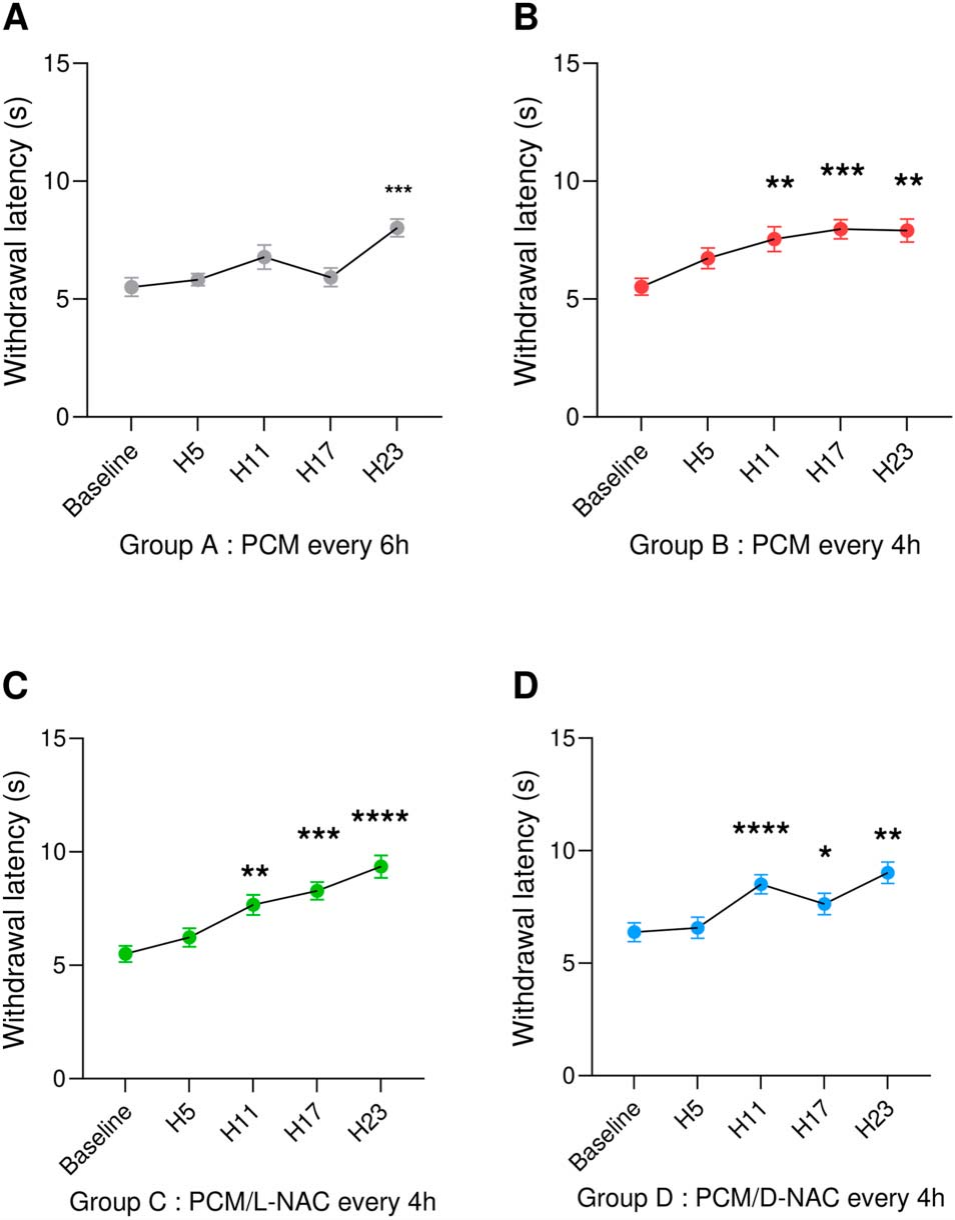
Time course in paw withdrawal latencies (plantar test) for the 4 treatment groups. (A) Group A (n = 18): 100 mg/kg of paracetamol (PCM), every 6 hours. (B) Group B (n = 19): 100 mg/kg of PCM, every 4 hours. (C) Group C (n = 19): 100 mg/kg of PCM + 100 mg/kg of N-acetyl-l-cysteine (L-NAC), every 4 hours. (D) Group D (n = 18): 100 mg/kg of PCM + 100 mg/kg of N-acetyl-d-cysteine (D-NAC), every 4 hours. Friedman tests: group A, *P* < 0.01; group B, *P* < 0.01; group C, *P* < 0.0001; group D, *P* < 0.001. Wilcoxon signed-rank tests for matched samples (vs. baseline): *P < 0.05; **P < 0.01; ***P < 0.001; ****P < 0.0001. Data are presented as mean ± SEM. H, hours post-treatment.

Table 1 and Figures 2 and 3 illustrate paw withdrawal latencies across the 4 treatment groups. Before treatment, there were no statistically significant differences in paw withdrawal latencies among the groups, indicating nociceptive homogeneity (Kruskal–Wallis test; *P* > 0.05; Fig. 2A). In contrast, Friedman tests demonstrated that latencies were significantly prolonged over time after treatment (Friedman tests; group A, *P* < 0.01; group B, *P* < 0.01; group C, *P* < 0.0001; group D, *P* < 0.001; Fig. 3). Specifically, post-hoc tests confirmed that the analgesic effect manifested at different times depending on the treatment. Notably, group A, which was treated with a conventional PCM regimen, exhibited a significant increase in latency compared with baseline after a long-term delay of 23 hours post-treatment (Table 1; Fig. 3A). Remarkably, the other 3 groups—group B (high-dose PCM), group C (PCM + L-NAC), and group D (PCM + D-NAC)—demonstrated significant analgesic efficacy as early as 11 hours postadministration (Table 1; Figs. 3B–D). Moreover, intergroup comparisons revealed a significant treatment effect on withdrawal latencies (Kruskal–Wallis test; *P* < 0.01; Fig. 2B). Subsequent post-hoc tests indicated that withdrawal latencies were significantly delayed for groups B, C, and D when compared with the conventional PCM regimen group (group A) 17 hours after treatment (group A vs group B, *P* < 0.01; group A vs group C, *P* < 0.01; group A vs group D, *P* < 0.05; Fig. 2B). Furthermore, the antinociceptive activity did not vary significantly among groups treated with the high dose of PCM, whether combined or not with L-NAC or D-NAC (group B vs group C, *P* > 0.05; group B vs group D, *P* > 0.05; group C vs group D, *P* > 0.05; Fig. 2B). The antinociceptive activity of the treatments was again comparable at 23 hours across groups (Kruskal–Wallis test; *P* > 0.05; data not shown), affirming the delayed effect of the conventional treatment (as observed in intragroup comparisons; Table 1; Fig. 3A). Collectively, these results demonstrate that a regimen of 6 PCM administrations per 24 hours (group B) yields earlier and superior analgesia compared with the standard regimen (group A; 4 PCM administrations per 24 hours; Table 1; Fig. 2). In addition, the combination of PCM + NAC administered 6 times over 24 hours results in earlier and enhanced analgesia compared with a standard schedule of 4 administrations per 24 hours (Table 1; Figs. 2 and 3). Finally, a similar analgesic effect was observed with L-NAC and D-NAC when combined with PCM (Table 1; Fig. 2B).

**Table 1:** Effects of drugs on paw withdrawal latency measured with the plantar test.

Group	Withdrawal latency (s)				
	Baseline	H5	H11	H17	H23
Group A	5.51 ± 0.39	5.82 ± 0.26	6.78 ± 0.51	6.20 ± 0.45	8.19 ± 0.39***
Group B	5.52 ± 0.35	6.87 ± 0.47	7.33 ± 0.45**	7.58 ± 0.45***	7.33 ± 0.44**
Group C	5.53 ± 0.36	6.24 ± 0.42	7.68 ± 0.50**	8.69 ± 0.42***	9.68 ± 0.37****
Group D	6.38 ± 0.35	6.73 ± 0.38	8.42 ± 0.51****	7.71 ± 0.42*	8.63 ± 0.46**

Results are expressed as means ± SEM. Wilcoxon signed-rank tests for matched samples *P < 0.05; **P < 0.01; ***P < 0.001; ****P < 0.0001 (vs baseline). Group A (n = 18): 100 mg/kg of paracetamol, every 6 hours; group B (n = 19): 100 mg/kg of paracetamol, every 4 hours; group C (n = 19): 100 mg/kg of paracetamol + 100 mg/kg of L-NAC, every 4 hours; group D (n = 18): 100 mg/kg of paracetamol + 100 mg/kg of D-NAC, every 4 hours.

D-NAC, N-acetyl-d-cysteine; H, hours post-treatment; L-NAC, N-acetyl-l-cysteine.

**Figure 4.**
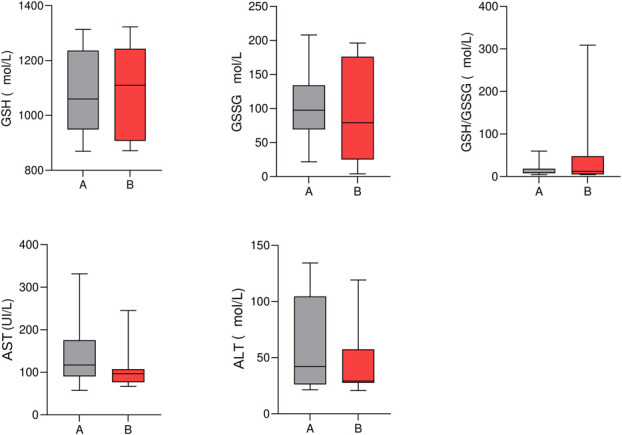
Liver biochemical markers among the 2 paracetamol (PCM)-treated groups. Group A (n = 14) was administered treatment every 6 hours (ie, 4 administrations of PCM at 100 mg/kg); group B (n = 15) received treatment every 4 hours (ie, 6 administrations of PCM at 100 mg/kg). Biochemical analyses were conducted 24 hours (GSH, GSSG) or 36 hours (AST, ALT) after treatment. Mann–Whitney U tests: *P* > 0.05 for all tests. Boxplots show distributions with black horizontal lines indicating the median, box margins denoting the lower and upper quartiles, and the whiskers extending from the box out to the most extreme observation within 1.5 times the interquartile range from the box. ALT, alanine aminotransferase; AST, aspartate aminotransferase; GSH, glutathione (reduced); GSSG, glutathione (oxidized).

**Figure 5.**
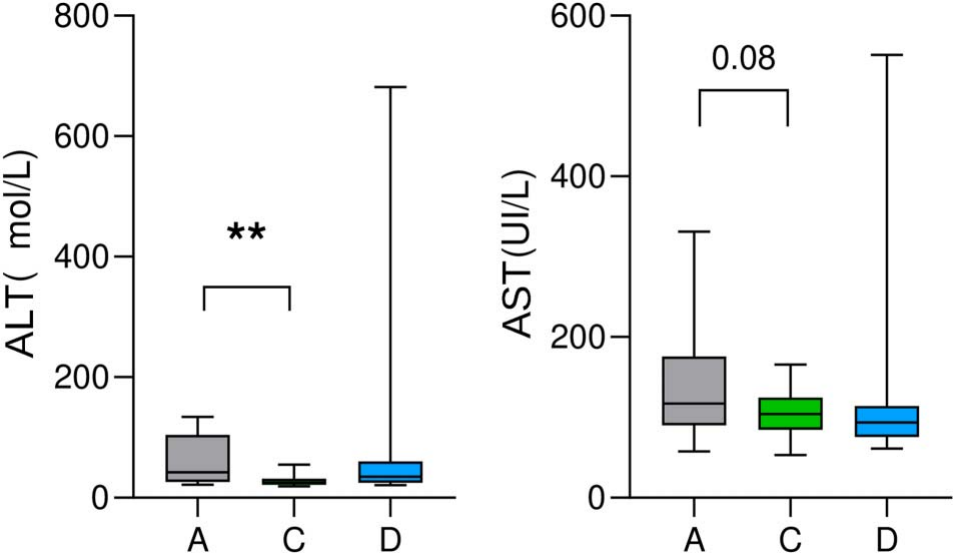
Effects of N-acetyl-l-cysteine (L-NAC) and N-acetyl-d-cysteine (D-NAC) on serum hepatic markers (ALT, AST) in paracetamol (PCM)-induced hepatotoxicity. Group A (n = 14): 100 mg/kg of PCM, every 6 hours; group B (n = 15): 100 mg/kg of PCM, every 4 hours; group C (n = 16): 100 mg/kg of PCM + 100 mg/kg of L-NAC, every 4 hours; group D (n = 17): 100 mg/kg of PCM + 100 mg/kg of D-NAC, every 4 hours. Kruskal–Wallis test: *P* < 0.05 (ALT). Dunn tests: **P < 0.01; *P* = 0.08. Boxplots show distributions with black horizontal lines indicating the median, box margins denoting the lower and upper quartiles, and the whiskers extending from the box out to the most extreme observation within 1.5 times the interquartile range from the box. ALT, alanine aminotransferase; AST, aspartate aminotransferase.

Table 2 and Figures 4 and 5 summarize the findings from biochemical analyses conducted across the different treatment groups. Before treatment administration, all groups exhibited comparable hepatic marker concentrations, with no statistically significant differences noted among them (Kruskal–Wallis test; *P* > 0.05; Supplementary Figure 1, http://links.lww.com/PR9/A434). This indicates that liver function was initially homogeneous among groups. To evaluate potential liver toxicity associated with high-dose PCM, we analyzed hepatic markers between the groups treated solely with PCM (group A, conventional PCM regimen; group B, high-dose PCM). The analysis revealed no significant changes in biochemical marker levels (Mann–Whitney U tests, *P* > 0.05; Fig. 4), suggesting that PCM administered every 4 hours (ie, 6 administrations) does not incur greater liver damage compared with a low dose (ie, 4 administrations every 6 hours). Notably, AST and ALT transaminase levels increased significantly after PCM treatment, irrespective of the administration regimen, in comparison to baseline (Table 2; Supplementary Figure 2, http://links.lww.com/PR9/A434). This increase was also evident after combined treatments of PCM with L-NAC or D-NAC (Table 2; Supplementary Figure 2, http://links.lww.com/PR9/A434). Importantly, the Kruskal–Wallis test indicated that L-NAC treatment significantly reduced transaminase levels (*P* < 0.05; Fig. 5). Post-hoc analyses confirmed that oral administration of L-NAC mitigated PCM-induced elevations in hepatic markers compared with the PCM-alone treated groups, particularly for ALT (Dunn tests; ALT: *P* < 0.01; AST:*P* = 0.08; Fig. 5; Supplementary Figure 3, http://links.lww.com/PR9/A434). Paracetamol did not significantly alter GSH at the H24 measurement point. The combination of D-NAC and PCM appeared to exhibit protective effects. Specifically, significant increases in GSH levels and decreases in GSSG levels, resulting in an elevated GSH/GSSG ratio, were observed (Table 2). However, this beneficial effect of D-NAC was not corroborated in comparisons between treatment groups (Kruskal–Wallis test, *P* > 0.05; data not shown). Overall, these findings suggest that a regimen of 6 PCM administrations per 24 hours (group B), as administered orally in our protocol, does not induce greater hepatic damage than the standard regimen (group A; 4 PCM administrations per 24 hours; Table 2; Fig. 4). Regarding transaminase level alterations induced by PCM, the combination of PCM + L-NAC administered in a schedule of 6 administrations per 24 hours offers significant hepatic protection (Table 2; Fig. 5; Supplementary Figure 3, http://links.lww.com/PR9/A434), with a reduction in transaminase levels exceeding 30% (ALT: group A vs group C, −48%; group B vs group C, −32%). Notably, for clinical relevance, a difference of at least 30% in transaminase activities between groups is deemed clinically significant.^27^ Finally, the protective effect of D-NAC combined with PCM on hepatic function is only suggested (Table 2).

**Table 2:** Effects of drugs on mouse hepatic function.

Group	GSH (baseline)	GSH (H24)	GSSG (baseline)	GSSG (H24)	GSH/GSSG ratio (baseline)	GSH/GSSG ratio (H24)
Group A	1070.2 ± 25.09	1083.9 ± 31.79	118.2 ± 11.73	105.7 ± 12.73	13.8 ± 3.72	17.8 ± 4.19
Group B	1068.3 ± 39.58	1085.6 ± 34.51	99.4 ± 12.54	96.6 ± 16.73	16.4 ± 3.11	57.5 ± 21.58*
Group C	1052.9 ± 33.17	1148.2 ± 27.88	94.4 ± 9.31	94.6 ± 10.69	15.2 ± 2.96	18.8 ± 3.83
Group D	1057.8 ± 19.82	1159.9 ± 27.85**	116.1 ± 8,70	83.0 ± 13.53*	10.3 ± 0.95	22.8 ± 3.71***

Group	GOT (baseline)	GOT (H36)	GPT (baseline)	GPT (H36)
Group A	59.5 ± 2.38	136.3 ± 16.04****	20.5 ± 3.06	58.7 ± 9.05***
Group B	61.3 ± 4.28	108.7 ± 11.01***	16.1 ± 1.05	43.6 ± 5.99****
Group C	62.9 ± 3.66	105.0 ± 6.51****	19.1 ± 1.60	28.5 ± 2.43**
Group D	54.8 ± 3.20	136.8 ± 31.25***	16.0 ± 1.05	112.4 ± 47.63****

Results are expressed as means ± SEM. Wilcoxon signed-rank tests for matched samples: *P < 0.05; **P < 0.01; ***P < 0.001; ****P < 0.0001 (vs baseline). Group A (n = 14): 100 mg/kg of paracetamol, every 6 hours; group B (n = 15): 100 mg/kg of paracetamol, every 4 hours; group C (n = 16): 100 mg/kg of paracetamol + 100 mg/kg of L-NAC, every 4 hours; group D (n = 17): 100 mg/kg of paracetamol + 100 mg/kg of D-NAC, every 4 hours.

D-NAC, N-acetyl-d-cysteine; GOT, glutamate oxaloacetate transaminase; GPT, Glutamate pyruvate transaminase; GSH/GSSG ratio, glutathione (reduced)/glutathione (oxidized) ratio; GSH, glutathione (reduced); GSSG, glutathione (oxidized); H, hours post-treatment; L-NAC, N-acetyl-l-cysteine.

## 4. Discussion

Paracetamol is extensively used in clinical settings to alleviate mild to moderate pain and/or to reduce fever. In the absence of rigorous adherence to prescribed dosages, an overdose of PCM can result in severe and irreversible hepatic damage. It thus represents the predominant cause of drug-induced liver transplants.^36^ N-acetylcysteine is recognized as the standard antidote for PCM toxicity. This agent acts as a precursor to glutathione, mitigating PCM's toxicity by augmenting hepatic glutathione levels, potentially through additional mechanisms that require further elucidation. Notably, it is remarkable that no combination of PCM and NAC has been commercialized to date aimed at enhancing the efficacy of PCM while minimizing its toxic effects. To address this apparent gap, we evaluated both the antinociceptive and hepatoprotective properties of the PCM-NAC combination within a murine model. The present findings reveal the advantages of combining NAC with PCM, particularly L-NAC, to augment both analgesic and hepatoprotective effects.

We used the plantar test, a sensitive method frequently used for thermal stimulation in rodents^6,14,33^ but also applicable to humans.^26^ First, our findings corroborate existing literature indicating that NAC does not diminish the therapeutic efficacy of PCM.^34^ More importantly, our results demonstrate a synergistic effect when L-NAC or D-NAC is combined with PCM administered every 4 hours. Indeed, this combination results in increased withdrawal latencies in the plantar test, exhibiting a superior analgesic effect compared with the standard PCM treatment. This observation aligns with previous studies on rats, which indicated that the concurrent administration of L-NAC with PCM dose-dependently enhanced the analgesic effects of PCM, as evidenced in the hot plate test.^28^

In addition, it is noteworthy that our results constitute the first report in the literature indicating that the administration of PCM every 4 hours produces an earlier analgesic effect compared with an administration frequency of every 6 hours; this effect persists throughout the chronic period. The observation that PCM administered alone does not elicit an analgesic response during the initial hours postadministration might appear surprising at first. However, we can exclude the possibility of habituation to the test, as mice receiving repeated doses of PCM combined with L-NAC or D-NAC exhibit earlier (from 11 hours postadministration) and sustained decreased sensitivity over time (up to 23 hours postadministration) to thermal nociceptive stimuli, indicative of the analgesic properties rather than mere behavioral adaptation to treatment. Our data suggest a cumulative analgesic effect of orally administered PCM in mice. Although the cumulative analgesic attributes of PCM have been documented across various pain models,^4,18,32^ specific evidence derived from the plantar test is less frequently reported compared with methodologies like the hot plate, formalin, or tail-flick tests. This cumulative effect is influenced by the dosage, timing, and frequency of administration. For instance, substantial doses of PCM are requisite to achieve measurable analgesia with the hot plate.^28,48^ Paracetamol's efficacy in the plantar test has been cited, particularly at elevated doses.^28,37^ Here, the dosage of 100 mg/kg per administration is supratherapeutic relative to the standard human therapeutic dose (∼15 mg/kg per administration), given that APAP pharmacokinetics are comparable between mice and humans, making direct mg/kg comparison more appropriate than allometric body surface area scaling.^19^ Although the Human Equivalent Dose calculated using the FDA body surface area conversion factor (100/12.3 ≈ 8.1 mg/kg) may suggest otherwise, this approach serves only as a starting point and does not supersede species-specific pharmacokinetic data when available.^12,35^ However, our dose remains substantially lower than those used in standard acute hepatotoxicity models (eg, 250–600 mg/kg, single i.p. injection).^2,4^ Combined with the oral route of administration and the repeated dosing schedule, this approach models chronic supratherapeutic exposure rather than acute overdose, as supported by the mild-to-moderate transaminase elevations observed without mortality. This is precisely the clinical context in which a preventive NAC coadministration strategy would be most relevant. Conversely, the cumulative analgesic effects of PCM are especially pronounced when the drug is administered at intervals (eg, 2–6 hours) conducive to maintaining effective plasma and tissue concentrations, thereby supporting the concept of pharmacodynamic accumulation^32^ and facilitating continuous central nervous system modulation of nociception. In alignment with this hypothesis, existing literature indicates that PCM operates centrally to alleviate pain, in contrast to NSAIDs, which primarily exert peripheral effects. These central actions of PCM may contribute to the cumulative effects observed over time, as receptor-mediated processes and neurochemical alterations are modulated upon repeated administration. Several studies have indeed demonstrated that PCM produces analgesic effects through central mechanisms, including the modulation of prostaglandin synthesis via inhibition of cyclooxygenase enzymes, activation of serotonergic and endocannabinoid pathways, and TRPV1 receptor desensitization.^4,11^ These mechanisms imply that repetitive dosing of PCM may amplify analgesic effects because of prolonged engagement with receptors and signaling pathways. More targeted studies focused on the plantar test are warranted to definitively ascertain the extent of cumulative behavioral and analgesic effects attributable to PCM.

We present here evidence of a comparable analgesic effect of L-NAC and D-NAC combination with PCM. Notably, L-NAC is the enantiomer of NAC administered in cases of PCM overdose.^41^ A recent review focusing on the use of L-NAC for managing chronic pain in patients indicated that NAC may confer analgesic efficacy for certain pain conditions. However, because of the absence of randomized controlled trials addressing this specific issue, it remains inconclusive regarding the efficacy of L-NAC in pain management.^25^ In a French study involving healthy volunteers, PCM was administered over 4 days (1 g administered 4 times daily) alongside L-NAC or a placebo.^29^ The antinociceptive effect of PCM was found to be comparable in both groups. Nevertheless, GSH levels were maintained at baseline in the PCM-NAC group, whereas they decreased in the PCM-placebo group.^34^ This capacity to sustain effective pain relief while potentially diminishing the risk of liver damage renders this combination particularly promising for clinical applications. Given these findings, it was crucial to quantify various markers of liver integrity in our preclinical model.

To investigate the toxicity profile resulting from escalating doses of PCM, as well as the potentially advantageous effects of the NAC-PCM combination, we quantified standard markers of liver function, including GSH, GSSG, AST, and ALT. A key limitation is the assessment of glutathione status via whole blood. This nonlethal method was chosen to facilitate longitudinal analysis, an approach supported by data correlating circulating and hepatic glutathione levels.^40^ We also acknowledge that GSSG quantification accuracy can be influenced by ex vivo oxidation. Although all samples were processed identically to maintain internal validity, future studies would benefit from incorporating preanalytical thiol stabilization techniques to improve the accuracy of absolute GSSG values.^24^ Furthermore, we acknowledge that direct measurement of hepatic GSH in vivo was not performed in our study. Such measurement, ideally at early time points (1–2 hours postdose) to capture the acute GSH nadir, would be necessary to confirm that the combination with NAC prevents hepatic glutathione depletion. However, this would have required animal sacrifice, precluding the longitudinal paired design central to our study. Similarly, liver histology was not performed but would have provided complementary morphological evidence regarding the degree of hepatocellular injury and the extent of protection afforded by NAC. These assessments should be prioritized in future studies. Although we hypothesized that more frequent dosing of PCM (6 administrations every 4 hours) would precipitate greater liver damage in comparison to the standard dosing regimen (4 administrations every 6 hours), the biochemical values did not consistently corroborate this hypothesis. This discrepancy can be readily attributed to the route of PCM administration. Indeed, PCM can be administered via intraperitoneal (i.p.) or intravenous (i.v.) injection, or by gavage (which we used). Numerous researchers opt for i.p. or i.v. administrations because of their superior reproducibility.^27^ Furthermore, PCM overdose (eg, 250 mg/kg body weight up to 500 mg/kg body weight) serves as a common experimental model in rodents to replicate acute liver failure observed in humans.^27^ In our experiments, we used gavage and avoided lethal doses of PCM (which did not result in fatalities during our study) to approximate the human therapeutic condition as closely as possible. However, it is acknowledged that this route of administration presents complexities because of individual variations in enteral absorption, which can lead to increased variability in the onset of liver injury. To mitigate this limitation, a larger cohort of mice and higher doses of PCM may have been warranted in our study. In addition, it has been demonstrated that enzymatic activities (AST, ALT) are considerably lower at lower doses of PCM (200 mg/kg) compared with higher doses (300 mg/kg).^21^ Strikingly, this deviation from our initial hypothesis ultimately reinforced our subsequent findings using NAC via the same route of administration. Indeed, we were able to demonstrate that the combination of L-NAC with PCM induced hepatic protection, as evidenced by reduced levels of transaminases, particularly ALT, a critical marker of liver injury. These results align with the hepatoprotective effects of L-NAC reported in the literature. For instance, PCM administration in mice induces liver damage in a dose-dependent manner (up to 600 mg/kg), an effect that can be reversed with the coadministration of L-NAC.^36^ In contrast, the protective effects of D-NAC on the liver were not observed to the same extent as those of L-NAC. No significant reduction in hepatic transaminase levels was found when D-NAC was combined with PCM. Conversely, our data suggest a potential beneficial effect on glutathione levels; however, these findings should be interpreted with caution (only intragroup statistical differences) and necessitate further investigation. The possibility of autophagy dysfunction represents a critical consideration for the safety profile of any high-frequency PCM regimen in susceptible individuals.^30^ However, NAC offers protection by preventing GSH depletion, which in turn blocks the initial formation of toxic adducts. It has been posited that glutamatergic neurotransmission is involved in pain transmission,^17^ and NAC has been shown to modulate the glutamatergic system.^31^

## 5. Conclusion

Our study demonstrates, for the first time in the literature, the synergistic benefits of the coadministration of NAC and PCM. Specifically, we show that both L-NAC and D-NAC enhance analgesic efficacy while concurrently reducing the hepatotoxicity associated with PCM in murine models. Furthermore, our data suggest that L-NAC may serve as a superior candidate compared with D-NAC for this indication, particularly in relation to its hepatoprotective effects. Further human trials of the NAC–PCM combination are warranted, contingent upon appropriate dosing strategies and safety protocols.

## Disclosures

The authors have no conflict of interest to declare.

## Supplemental digital content

Supplemental digital content associated with this article can be found online at http://links.lww.com/PR9/A434.
